# Maternal Mental Health Status Is Associated with Weight-Related Parenting Cognitions, Home Food Environment Characteristics, and Children’s Behaviors

**DOI:** 10.3390/ijerph192113855

**Published:** 2022-10-25

**Authors:** Melissa Keresztes, Colleen L. Delaney, Carol Byrd-Bredbenner

**Affiliations:** Nutritional Sciences Department, School of Environmental and Biological Sciences, Rutgers, The State University of New Jersey, New Brunswick, NJ 08901, USA

**Keywords:** mothers, depression, anxiety, stress, weight, cognitions, behaviors, home food environment, children

## Abstract

Women experience anxiety, depression, and stress at higher levels than men and have more parenting responsibilities, especially establishing health practices in the home. Given children’s vulnerability, this study aimed to increase understanding of how mothers’ mental health status relates to maternal weight-related cognitions, home food environments, and child health via a cross-sectional survey design. In a cluster analysis, using maternal anxiety, depression, and stress assessments, we placed the sample of 531 mothers of school-age children into four clusters: Cluster 1 had the best mental health status, Cluster 2 had high stress, Cluster 3 had anxiety and moderate stress, and Cluster 4 had anxiety, depression, and high stress. Our results indicate an overall downward trend in weight-related cognitions as mental health worsened. Similarly, as mental health declined, so did home food environment characteristics, such as the greater use of non-recommended child feeding practices, fewer family meals, and greater sugar-sweetened beverage supplies. As mothers’ mental health status became poorer, children’s general health and mental health quality of life declined, and sugar-sweetened beverage intake increased. Our findings suggest that maternal stress, anxiety, and depression are moderately to strongly linked with mothers’ cognitions, home food environments, and children’s health. Our results also suggest that mental health interventions for mothers should assess cognitions and home food environments and consider the extent to which these factors are affecting family health.

## 1. Introduction

Mental health disorders are prevalent in the United States [1,2], with nearly 52 million, or 1 in every 5 adults, experiencing a mental illness in 2019 [3]. Of these, 32 million, or more than 60%, were women [3].

Generalized anxiety disorder (GAD) and major depressive disorder (MDD) are two of the most common mental health disorders. The fifth edition of the *Diagnostic and Statistical Manual of Mental Health Disorders* (DSM-5) defines GAD as excessive worry and anxiety over various events or activities [4,5]. Symptoms of GAD include trouble sleeping, muscle aches, irritability, increased fatigue, restlessness, and difficulty with concentration [4,5]. Statistics from epidemiological studies suggest that women have a two to three times higher lifetime probability of GAD than men [6,7].

MDD is defined in the DSM-5 as a loss of interest or pleasure in daily routines [5]. Individuals with MDD may experience a decline in appetite, pleasure, sleep, energy, concentration, and/or motivation [5]. MDD is the leading cause of disease-related disability in women [8]. Depression is much more prevalent in women than in men, with a female to male ratio of approximately 2:1 [8]. Ten to 25% of adult women will experience a severe depression period during their lifetime, a rate lower than the 5 to 12% for men [9]. According to a cross-sectional analysis of 8915 mothers, 1 in 10 mothers suffered from depression during a 12-month span [10].

Long-term stress, although not considered a mental illness, is associated with poorer mental and physical health [11]. According to stress and coping theory, stress is an imbalance between one’s perceived demands and the personal/social resources accessible to meet those demands [11,12,13]. Stress is caused by life events, such as job loss, emotional and physical abuse, divorce, and parenting responsibilities [11,12,14]. Women report higher stress levels than men, especially stress related to caring for children [15,16,17,18,19]. Parental stress involves the physiological, emotional, social, and economic demands and pressures of being a parent [16]. Sex differences in parenting stress levels often stem from the increased responsibilities that mothers tend to have in relation to caring for children and associated time and financial limitations, as well as marital conflicts [20].

Depression and anxiety frequently go untreated in mothers which, in turn, may negatively affect children. For example, toddlers and preschoolers whose mothers are depressed may be less independent, less likely to interact with others, have trouble accepting discipline, be more aggressive, and have poorer physical health, including a greater risk of childhood obesity, which may be the result of poor eating habits [21]. Maternal depression and anxiety are associated with increased intrusive behaviors, such as the rough handling of children; less affection and emotional warmth; decreased interactions with their child; use of forceful, indulgent, or other non-recommended child feeding styles; fewer positive feelings of parenting skills; and a greater risk of mental illness in children [21,22,23,24,25]. In addition, maternal stress, depression, and anxiety are associated with poorer maternal physical health, including higher BMIs and a greater incidence of weight-related diseases [26,27].

Many mental health conditions are comorbid with obesity [28]. Although obesity affects both men and women, it is more prevalent among women at a rate of 40% compared to 35% in men [29,30]. Recent studies suggest a bidirectional association between obesity and depression as well as between obesity and anxiety [28,31,32,33]. Additionally, stress is associated with the development and maintenance of obesity [34,35,36,37].

Health-compromising behaviors are more common among individuals living with stress, depression, and anxiety [38,39,40,41,42,43]. For instance, women with greater stress, depression, and/or anxiety are likely to consume less fruits and vegetables, eat more fatty and sugary foods, engage in more emotional eating, drink more alcohol, get less exercise, and have a poorer sleep quality [38,43,44,45,46,47,48]. These unhealthy behaviors not only have deleterious effects on the health and weight of these women, but they also have the potential to negatively impact others in the family—notably children. However, little is known about how mothers’ mental health status is linked with their weight-related cognitions and the health and weight-related behaviors in children. Weight-related cognitions are thoughts, beliefs, and understandings (mental processes) that result in intentions to perform, or the actual performance of, behaviors associated with body weight status. For instance, understanding or believing that engaging in a particular behavior (e.g., eating fruits and vegetables) will have a desired outcome on body weight increases the likelihood of engaging in that behavior [49,50]. Other cognitions that affect the likelihood of engaging in a behavior include having confidence in one’s ability to perform the behavior, valuing the behavior and its outcome, and perceived support from others [49,50].

It is evident that women experience anxiety, depression, and stress at higher levels than men and also have greater risk of obesity. Additionally, women have more parenting responsibilities, especially with regard to establishing health practices in the home, such as deciding on the types of foods to buy and preparing and encouraging healthy behaviors in children [51]. Given the vulnerability of children, it is important to gain a better understanding of how mothers’ mental health status is related to child health and weight-related behaviors. This study hypothesized that mothers with poorer mental health status would exhibit more negative weight-related parenting cognitions and home food environment characteristics than mothers with better mental health status. In addition, children of mothers with poorer mental health status were hypothesized to engage in more unhealthy behaviors and, therefore, have worse health and a higher body mass index (BMI) than their counterparts whose mothers have better mental health status. Because limited research has examined the mental health status of mothers with children in age groups older than infants and toddlers, this study targeted mothers of school-age children. The results of this study can provide insights for the design of future interventions for mothers with poor mental health status to improve both their health and BMI status and that of their children.

## 2. Materials and Methods

This cross-sectional survey was approved by the Institutional Review Board at the authors’ university. All participants gave informed consent by clicking the “agree to participate” button prior to completing the online study survey. The study reported here is a secondary analysis of data from the cross-sectional Home Obesogenicity Measure of EnvironmentS-2 (HOMES-2) [52].

### 2.1. Sample

The eligibility criteria included being between 25 and 54 years of age, a mother of at least one child between the age of 6 to 11 years, and the primary household food gatekeeper (i.e., the person who makes all or most of the food purchasing and preparation decisions). Eligible mothers also had to be able to read English, have access to the internet, and be residents in the United States.

Dynatec (dynata.com), one of the world’s largest data organizations, recruited a non-probabilistic sample of mothers for the survey during March 2020. Mothers received a modest stipend equivalent to about $10 USD for their time spent completing the survey and were paid in the form of either cash, prizes, points, and/or charitable contributions. The sample size was estimated based on a 95% confidence level and the total population of women in the United States between the ages of 25–54 years old to ensure that the population characteristics were adequately estimated by the survey. Recruitment continued until the sample size equaled or exceeded the minimum sample size of 385. Of the 1342 mothers who were recruited, 531 met all the eligibility criteria, gave informed consent, and completed the survey.

The study participants had an average age of 37.54 ± 5.84 SD years and lived in regions spread across the United States, mirroring the United States population distribution [53]. The sample was more diverse than the U.S. population of women in the age group: fewer were White (35% vs. 57%), more were Black (20% vs. 14%), and more were Asian (15% vs. 8%), while the percentages were similar for Hispanic (25% vs. 26%) and American Indian/Alaskan Natives/Native Hawaiian/other Pacific Islanders (1% for both) [54,55]. The mothers’ education level was comparable to national data for women in the U.S. in that 9 out of 10 had a high school education or higher [56]. A total of 67% of participants in this study were in dual parent households, which is similar to the 65% national average [55]. Additionally, the participants had an average of 2 children under age 18 years living in the household, which is the same as the national average [57]. At 12%, the percent reporting food insecurity was comparable to national data on households reporting food insecurity [58].

### 2.2. Instruments

The data collected included assessments of maternal mental health, sociodemographic characteristics, weight-related parenting cognitions, and home food environment characteristics, as well as the health status and weight-related behaviors of school-age children [52]. Mothers with more than one child between the targeted age range of 6 to 11 years were instructed to report on the child born closest to a predetermined randomly selected date and time.

Given the prevalence of anxiety, depression, and stress in women and the availability of valid, reliable self-report questionnaires, these conditions were used to assess maternal mental health. The Generalized Anxiety Disorder (GAD)-2, Patient Health Questionnaire (PHQ-2), and Stress Load subscale of Cohen’s Perceived Stress Scale-4 (PSS-4) were used to assess anxiety, depression, and the stress load, respectively [15,59,60,61,62,63,64]. All 3 of these widely used scales each contained 2 items that determined the frequency of symptoms of the mental health condition in the past 2 weeks using 4 answer choices (i.e., not at all, several days, more than half the days, nearly every day), which were scored from 1 to 4, respectively. The items on each scale were averaged for a final score. Higher scores on each scale indicated a greater frequency of symptoms.

The sociodemographic characteristics included the mother’s age, education level, and ethnicity/race, and the number of parents and children under the age of 18 years living in the household. Food insecurity risk was assessed with Hager et al.’s 2-item scale (e.g., “In the last year, the food I bought just did not last and we did not have money to get more”), which is scored using a 4-point Likert scale with the following responses: definitely false, mostly false, mostly true, and definitely true, respectively. Item scores were averaged, with higher scores suggesting greater food insecurity risk [65].

Weight-related parenting cognitions were assessed with 6 scales from the HOMES-2 survey (see Supplementary Materials 2 in Byrd-Bredbenner et al. [52]). Details of the development and content of each scale are reported elsewhere [52]. In brief, the scale items had the 5-point-Likert-type answer choices of strongly disagree, disagree, neither agree nor disagree, agree, and strongly agree, which were scored from 1 to 5, respectively. Scale scores were determined by averaging the item responses. Higher scores indicated a more positive expression of the cognition and, therefore, a greater likelihood of performing the behavior associated with the cognition [49,66,67]. The cognition scales assessed *Self-efficacy for Promoting Healthy Child Weight-Related Behaviors* (13 items, Cronbach alpha = 0.91; sample item: “I am confident that I will get my school-age kids to be active and burn off energy for a total of at least 60 min every day”), *Importance Placed on Modeling Healthy Weight-Related Behaviors for Children* (7 items, Cronbach alpha = 0.86; sample item: “It is important that my school-age kids are aware that I eat fruits and vegetables several times each day”), *Importance Placed on Healthy Weight-Related Behaviors for Children* (13 items, Cronbach alpha = 0.87; sample item “It is important that my school-age kids get enough sleep every night to wake up rested”), *Encouragement and Facilitation of Children’s Healthy Weight-Related Behaviors* (12 items, Cronbach alpha = 0.84; sample item “Over the past 2 weeks, I often encouraged my school-age kids to decide how much healthy foods to eat at meals”), *Maternal Perception that Engaging in Healthy Weight-Related Behaviors is Worth the Effort* (12 items, Cronbach alpha = 0.97; sample item “It is not worth the effort to get my school-age kids to eat breakfast”), and *Family Support for Healthy Weight-Related Behaviors* (11 items, Cronbach alpha = 0.94; sample item: “In the last 2 weeks, my family complained about having to limit sugary drinks”; reverse-scored).

The weight-related characteristics of the home food environment evaluated were maternal child feeding practices, household food availability, and family mealtime patterns. Three scales assessed the child feeding practices of mothers: pressuring children to eat nutrient-dense food, restricting children’s access to low-nutrient-dense foods, and rewarding children for eating healthy foods (i.e., instrumental feeding). Each scale had 2 items with the 5-point-Likert-type answer choices of never, rarely, sometimes, most of the time, and always, which were scored from 1 to 5, respectively. Items on each scale were averaged to create the scale score, with higher scores reflecting more frequent use of these non-recommended child feeding practices [52].

The HOMES-2 Fruit/Vegetable Availability 7-item scale assessed the servings of fruits and vegetables available per person in the household each day [68,69,70]. The 1-item HOMES-2 Sugar-Sweetened Beverage Availability scale assessed the number of servings available per person per day of sugar-sweetened beverages [52]. The HOMES-2 survey Breakfast Food Availability indicator item assessed the household availability of breakfast foods using 5-point-Likert-type answer choices ranging from strongly disagree to strongly agree (scored from 1 to 5, respectively) with regard to the respondent’s perception of having enough breakfast foods available for family members to eat breakfast each day [52]. The aspects of family mealtime patterns assessed were the total number of meals eaten together as a family each week and the total number of meals eaten in unhealthy settings (i.e., in cars, at convenience stores or fast food restaurants, and while using electronic media) [52].

Child health status was assessed using items from the CDC’s Health-Related Quality of Life questionnaire [71]. Mothers rated children’s general health using a 5-point scale ranging from poor to excellent, scored from 1 to 5. They also reported the number of days in the past month in which children had “not good” physical and mental health [71]. Children’s BMI percentile was calculated using the child heights and weights reported by the mothers. The 7-item Block Fruit/Vegetable Screener [68,72,73] determined the servings/day of fruits and vegetables and the 1-item HOMES Sugar-Sweetened Beverages Frequency measured the daily servings of sugar-sweetened beverages [52]. The Streamlined, Enhanced Self-Report Physical Activity Measure measured the physical activity level, which could range from 0 to 49 [74]. The sleep duration (hours/night) and sleep quality (5-point scale ranging from very bad to very good) were measured using the Pittsburgh Sleep Quality Index [75,76].

### 2.3. Data Analysis

The mothers were assigned to mental health status groups by cluster analyzing their responses to the following variables: the GAD-2, PHQ-2, and PSS-4 Stress Load subscale scores. Cluster analysis is used to merge individuals into meaningful groups, accounting for similarities within groups while simultaneously creating unique groups that are different from each other [77,78]. A 3-step clustering procedure was used. In step 1, Ward’s Hierarchical Cluster Analysis was conducted to determine the ideal number of clusters by examining the scree plot and agglomeration schedule in order to determine the point at which the difference between clusters sharply increased [78]. In step 2, a k-means cluster analysis was conducted using the ideal number of clusters identified in step 1, followed by analysis of variance (ANOVA) and Tukey’s post hoc procedures for all the pairwise comparisons of the clustering scales. In step 3, the cluster stability was established by repeating step 2 11 times (2% of the sample size) using a different, randomly selected half of the sample each time. Cohen’s kappa coefficients were calculated to compare the agreement between the original k-means cluster assignment and the assignment generated for each k-means cluster using the 11 different, random halves of the sample. The 11 Cohen’s kappa coefficients were averaged, and a standard deviation was computed.

ANOVA and Tukey’s post hoc tests were conducted to compare the maternal mental health clusters with regard to each sociodemographic characteristic, weight-related cognition, home food environment characteristic, and child health status and behaviors. Significance was set at *p* < 0.05. Partial eta-squared values were calculated to indicate the effect sizes of significant ANOVA comparisons, with effect sizes of 0.01, 0.06, and 0.14 indicating small, medium, and large affects, respectively [79]. The cluster analysis procedures were completed using JMP Pro version 16.1.0 (SAS Institute, Inc., Cary, NC, USA), with all other analyses conducted using SPSS software version 28 (IBM Corporation, Chicago, IL, USA).

## 3. Results

### 3.1. Cluster Analysis

Our examination of the scree plot and agglomeration schedule from Ward’s cluster analysis of the GAD-2, PHQ-2, and PSS-4 Stress Load subscale scores revealed a sharp increase, or “elbow”, in the scree plot at stage 527. The ideal number of clusters was determined by subtracting the stage of the elbow (527) from the total sample size (i.e., 531), and this indicated that there were four ideal clusters. Then, the four-cluster k-means analysis was conducted and, as shown in Table 1, the ANOVA revealed significant differences in the mean scores for all three clustering scales, with large effect sizes. Tukey’s post hoc tests findings revealed that all the pairwise cluster comparisons were significantly different, thereby indicating that each cluster represented a unique grouping. Cohen’s kappa coefficients, comparing the agreement of the cluster assignment between the original cluster assignment and each of the 11 repeated k-means clusters of the random halves, averaged 0.82 + 0.12 SD, reaching a level of agreement that meets the threshold set by Landis and Koch of “almost perfect” [80], thereby confirming the cluster stability.

As the cluster assignment number increased from 1 to 4, the mean clustering scale scores tended to increase. Cluster 1 mothers scored significantly lower on all clustering scales than all the other clusters. These mothers experienced stress, symptoms of depression, or symptoms of anxiety least frequently. Cluster 2 mothers scored significantly lower than Clusters 3 and 4 on the depression and anxiety scales. Cluster 2′s stress load was significantly lower than that of Cluster 4 but higher than that of Cluster 3. Thus, Cluster 2 experienced the third most frequent symptoms of anxiety and depression but had higher stress than all the clusters except Cluster 4. Cluster 3 scored significantly lower than Cluster 4 on all the clustering scales. Cluster 3 mothers had the second highest frequency of depression and anxiety symptoms but had only the second highest stress load. Cluster 4 scored significantly higher on all the clustering scales than all the other clusters. These mothers most frequently experienced a high stress load and symptoms of depression and anxiety.

### 3.2. Sociodemographic Characteristics

Table 2 reports that maternal age tended to decline as the cluster assignment increased, with mothers in Cluster 1 being significantly older than those in Cluster 4. However, the effect size was small. Cluster 3 had a significantly smaller proportion of White mothers than Clusters 2 and 4, but the effect size was small. The average education level was at least some post-secondary education fin all the clusters, but mothers in Cluster 4 had significantly less education than Clusters 1 and 2. However, the effect size was small. Most mothers were in dual parent households and had an average of 2 children, with no between-cluster differences. The mothers experienced some food insecurity, with significant differences between all the pairwise comparisons, showing a large effect size. Mothers in Cluster 1 had significantly less food insecurity risks than all the other clusters, whereas Cluster 4 had significantly more.

### 3.3. Maternal Weight-Related Parenting Cognitions

Table 3 indicates and overall downward trend in the weight-related cognition scale scores as the cluster assignment number increased. Cluster 1 scored significantly higher than all the other clusters on all the parenting cognition scales. The ANOVA and Tukey’s post hoc results for the *Self-Efficacy for Promoting Child Healthy Weight-Related Behaviors Scale* indicate that all the mothers felt confident in encouraging their children to perform healthy weight-related behaviors, with mothers in Cluster 1 having significantly more confidence than all the other clusters. The *Importance Placed on Modeling Healthy Weight-Related Behaviors Scale* scores revealed that mothers tended to agree that it was important for them to model healthy weight-related behaviors; however, Cluster 1 mothers placed significantly greater importance on modeling than Clusters 3 and 4. The *Importance Placed on Healthy Weight-Related Behaviors for Children Scale* results showed that mothers in Cluster 1 scored significantly higher than all the other mothers. Comparisons of the average scores for the *Encouragement and Facilitation of Children’s Healthy Weight-Related Behaviors Scale* showed that the mothers tended to agree that they encouraged children, with Cluster 1 giving children significantly more encouragement than all the other clusters. The average scores for the *Perception that Engaging in Healthy Weight-Related Behaviors is Worth the Effort Scale* tended to differ significantly between all pairwise clusters, with Cluster 1 scoring the highest, followed by Cluster 3, Cluster 2, and Cluster 4, with a large effect size. The *Family Support for Healthy Weight-Related-Behaviors Scale* scores declined as the cluster group assignment increased, with all clusters differing significantly from each other and having a large effect size.

### 3.4. Home Food Environment

Table 4 shows that for the *Pressuring Children to Eat Nutrient-Dense Foods Scale*, Cluster 1 mothers disagreed that they used this negative child feeding practice. Other clusters were more neutral about their use of this practice, with Cluster 4 slightly agreeing that they used it. All clusters tended to differ significantly from each other, with a large effect size. The results of the *Restricting Children’s Access to Low-Nutrient-Dense Foods Scale* revealed that Cluster 1 mothers somewhat disagreed that they used this negative feeding practice, whereas mothers in other clusters somewhat agreed that they engaged in this practice, with all the pairs tending to differ significantly from each other, revealing a large effect size. The *Using Instrumental Feeding Strategies Scale* scores indicated that mothers in Clusters 1 and 3 tended to disagree that they rewarded children with food, whereas Cluster 2 and 4 mothers had higher scores. The ANOVA and Tukey’s post hoc tests revealed that Cluster 1 tended to score significantly lower than the other clusters, with a medium effect size.

As the cluster group number increased, the home availability of fruits and vegetables and breakfast foods tended to decrease, while sugar-sweetened beverages tended to increase. All the clusters had about five servings of fruits and vegetables and less than one serving of sugar-sweetened beverages available per household member per day. Cluster 4 scored significantly lower on the fruit/vegetable availability scale and higher on the sugar-sweetened beverage availability scale than Cluster 1; however, the effect size was small. All clusters agreed that there was sufficient breakfast foods available for all family members to eat breakfast daily; however, Cluster 1 tended to score significantly higher than all the other clusters, with a small effect size.

Mothers in Cluster 1 had significantly more family meals weekly than Clusters 2 and 4, with a small effect size. Cluster 4 mothers reported significantly more meals in unhealthy locations than all the other clusters, with a medium effect size.

### 3.5. Child Health and Weight-Related Behaviors

As shown in Table 5, the children had very good health; however, Clusters 3 and 4 mothers reported significantly poorer health for their children than those in Cluster 1. The mothers reported children had about 1 day per month of poor physical health and less than 3 days per month of poor mental health; however, mothers in Clusters 3 and 4 reported significantly more days of poor mental health for their children than mothers in Clusters 1 and 2, with a medium effect size.

The children in all the clusters had a BMI percentile indicative of a healthy weight. They consumed an average of four servings of fruits and vegetables per day. Although all the children had low intakes of sugar-sweetened beverages, children in Cluster 4 had significantly more sugar-sweetened beverage servings daily than all the other clusters, with a medium effect size.

The children of mothers in all the clusters had low physical activity, achieving less than 50% of the possible 49 points on this scale. The mothers in all but Cluster 4 reported that their children slept for the recommended 8.5 to 9 h per night [81]. The children’s sleep quality was very good; however, children in Cluster 1 had significantly better sleep quality than children in Cluster 3 and Cluster 4.

## 4. Discussion

This study examined the relationships between the mental health status of mothers of school-age children, as indicated by anxiety, depression, and stress symptom frequency, and their weight-related parenting cognitions, home food environments, and their children’s health status and weight-related behaviors. The mothers were clustered into four mental health status groups: Cluster 1 mothers experienced symptoms of depression and anxiety least frequently, with the symptom frequency increasing progressively from Cluster 2 to Cluster 3 and again to Cluster 4. The stress load followed a slightly different trend in that Clusters 1 and 4 had the lowest and highest stress, respectively, as was also the case for anxiety and depression, but Cluster 2′s stress load was higher than that of Cluster 3.

The threshold score indicative of MDD is ≥3 on the PHQ-2 [59,60,61,82], and a clinically significant GAD is indicated by a score of ≥2 on the GAD-2, with a score of ≥3 recommended by other authors [62,83,84]. The threshold indicative of a high, moderate, and low stress load, extrapolated from the 10-item version of the PSS, is ≥68%, >35 and <68%, and ≤35% of the total possible score, or ≥2.72, >1.4 to <2.72, and ≤1.4 points for the four-point Stress Load subscale of the PSS-4 [85,86]. The mothers in Cluster 4 met the thresholds for MDD, GAD, and a high stress load. Those in Cluster 3 met the thresholds for GAD and a moderate stress load. Cluster 2 mothers met the threshold for a high stress load. Cluster 1 did not meet the thresholds for any of the mental health status measures. Thus, Cluster 1 had the best mental health status, Cluster 2 had high stress, Cluster 3 had anxiety and moderate stress, and Cluster 4 had the worst mental health, with high anxiety, depression, and stress.

The 100 mothers in Cluster 4, or about 19% of the sample, reached the threshold suggesting MDD—a rate nearly twice as high as the 10.5% national average for women [87]. In addition, more than 40% of the mothers (Clusters 3 and 4) reached the lower threshold (i.e., a ≥2 score) for GAD—higher than the 19% national average for women [88]. However, the mothers in Cluster 4 met the ≥3 GAD threshold—the same prevalence rate as the national figures. These assessments used for MDD and GAD (i.e., PHQ-2 and GAD-2) are screeners that indicate that a follow-up by health care professionals should be conducted to ascertain whether a diagnosis of MDD or GAD is warranted. However, these high percentages are worrisome, given the associations between mental health and long-term health outcomes and parenting [14,89,90,91,92,93].

The only sociodemographic characteristic that differed significantly between clusters and had an effect size reflective of a strong relationship with mental health status was food insecurity. Research suggests that mothers experiencing food insecurity are at a greater risk of symptoms of mental illness than food-secure mothers [94], findings that are supported by this study. That is, symptoms of GAD, MDD, and stress tended to increase in tandem with food insecurity risk, likely because of the worry, concern, and stress associated with the mothers’ lack of sufficient resources to adequately and appropriately feed their families. These findings suggest that mental health treatment facilities should screen patients for food security and offer solutions to ameliorate instances of food insecurity, such as referral to government food and nutrition education programs and/or local resources (e.g., food pantries). Additionally, programs aiming to improve food security should consider offering mental health screening opportunities and referrals.

The hypothesis that mothers with poorer mental health status would exhibit more negative weight-related parenting cognitions was borne out by the study findings. As mothers’ mental health status declined, their weight-related cognitions trended downward significantly, with moderate to strong relationships between these variables. These findings are of particular concern given that the cognitions assessed are all associated with the likelihood of performing the weight-protective behaviors assessed (e.g., promoting healthy behaviors in children, modeling these behaviors, encouraging children to perform these behaviors, and valuing weight-related parenting behaviors), and the environment needs to be supportive of the behaviors to be performed [49,50,67].

The results of this study also show support for the hypothesis that mothers’ mental health status and home food environment characteristics would differ in the same direction. That is, as maternal mental health declined, so did the home food environment characteristics. Most differences in the home food environments had weak relationships, except for the use of non-recommended child feeding practices and frequency of family meals in unhealthy settings, which had moderate to strong relationships. Our home food environment findings support previous works reporting that women with poor mental health were more likely to display negative child feeding practices during mealtimes [23,95]. Congruent with the literature, mothers with the poorest mental health used more non-recommended child feeding practices in that they were more likely to pressure their children to eat nutrient-dense foods, restrict children’s access to low-nutrient-dense foods, and use more instrumental feeding practices, such as bribing and rewarding children to eat.

Frequent family mealtimes are associated with mental health benefits, including lower rates of anxiety and depression [96,97,98,99]. The findings of the current study align with the literature in that mothers with the most mental health symptoms tended to have fewer family meals in total and more family meals in unhealthy locations. Interestingly, the mothers in Cluster 2 reported less frequent family meals than all the other clusters, significantly differing from those with less stress alone (Clusters 1 and 3). Additionally, the most stressed clusters, Clusters 2 and 4, had significantly more family meals in unhealthy settings. These family meal results may suggest that stress may affect mealtime behaviors independently of other mental health conditions.

Maternal mental health is closely related to that of children in the family and can affect children’s development and behaviors [100,101]. Maternal mental health is linked to the inability to effectively or consistently parent, in turn contributing to negative weight-related parenting behaviors, including an increased likelihood of providing children with low-nutrient-dense foods and giving less encouragement at family mealtimes [16,23,95]. The current study findings align with the literature in that the children of mothers with poorer mental health had a greater availability of sugar-sweetened beverages in their homes, and these children drank more sugar-sweetened beverages despite their mothers reporting a greater likelihood of restricting children’s access to low-nutrient-dense foods.

Some evidence in this study reveals that the hypothesis that the children of mothers with poorer mental health status engaged in more unhealthy behaviors was true in that the children of Cluster 4 mothers drank more sugar-sweetened beverages, had an insufficient sleep duration, and had poorer-quality sleep. However, other behavioral measures (child physical activity level and fruit/vegetable intake) did not differ by maternal mental health status. BMI also did not differ between clusters, indicating a lack of support for the hypothesis that maternal mental health and child BMI were inversely related. Similar to previous research, as hypothesized, the current study findings show that as mothers’ mental health status became poorer, children’s general health and mental health quality of life declined [100,101]. Additionally, inadequate sleep and poor sleep quality are correlated with poorer mental health, which is consistent with the current study’s findings [4,5,47].

Trends in our study findings suggest that maternal stress, depression, and anxiety are moderately to strongly linked with mothers’ weight-related cognitions, some aspects of the home food environment, and children’s health and some behaviors. The results of this study imply that mental health interventions for mothers should assess cognitions and home food environments and consider the extent to which these factors are affecting family health. Offering mothers who are receiving mental health services strategies to improve their weight-related cognitions and reshape the home food environments could help to safeguard family health and reduce obesity risks. Similarly, it may be wise for nutrition education interventions targeting the parents most at risk of mental health conditions to help them to better understand how their own cognitions affect the health of the family by using instructional approaches aimed at improving cognitions (e.g., motivational interviews, observational learning opportunities), as well as providing parents with strategies for small, simple changes to the home food environment that would support better family health.

The results of this study, as in any study, need to be considered in light of its strengths and limitations. The cross-sectional nature of this study can only reveal associations between mental health and weight-related cognitions, home food environment characteristics, and child health and behaviors and cannot determine causality. Future research should aim to elucidate these temporal relationships. Another limitation is that the data were self-reported and, therefore, may be biased according to social desirability. However, the online data collection method allowed participants to complete the survey privately, a method which helps to reduce the social desirability bias [102,103]. Although mothers with more than one child in the school-age range reported on only a single child in the family, the selection bias was minimized by instructing mothers to report on the child born closest to a specified date and time. A further limitation is that the sample was comprised of mothers, and the results cannot be generalized to fathers and other partners or caregivers. However, the sample was large, racially diverse, and recruited from across the United States. The use of valid, reliable instruments and robust statistical procedures to conduct the cluster analysis and confirm the cluster stability are other important study strengths.

The results of this study can guide potential future interventions, health programs, and research. The maternal mental health characteristics found to be associated with poorer weight-related cognitions, home food environments, and child behaviors can be used to develop a prospective plan of action for minimizing negative health outcomes associated with greater mental health symptoms in mothers. In addition, future research should consider examining the relationships between the mental health and weight-related cognitions of mothers with school-age children in a longitudinal study to overcome the limitations of the cross-sectional nature of the current study. A greater understanding of the directionality of the relationships between mental health and other health cognitions and behaviors could be used to form potential prevention and treatment plans. Most importantly, early and continuous interventions should be considered for mothers reporting mental health symptoms, perhaps in healthcare settings where care is already being provided for the ease of both the parent and child, especially given that women who report symptoms of mental health early are likely to experience these symptoms again in the future [104].

## 5. Conclusions

To the best of the authors’ knowledge, this is the first study to examine the relationship of maternal mental health with weight-related cognitions, home food environment characteristics, and child health and behaviors. The study findings suggest that maternal mental health has strong links to maternal weight-related cognitions, home food environments, and child health and behaviors. Thus, maternal mental health status should be considered when assessing nutritional health and addressed in interventions seeking to improve nutritional health.

## Figures and Tables

**Table 1:** Comparison of Maternal Mental Health Status Clusters (N = 531).

Clustering Scale	Clusters				F Value df = 3, 527 #	ANOVA ^1,‡^ *p*	Partial Eta-Squared
	Cluster 1 Mean ± SD (CI *) n = 230	Cluster 2 Mean ± SD (CI) n = 83	Cluster 3 Mean ± SD (CI) n = 118	Cluster 4 Mean ± SD (CI) n = 100			
Perceived Stress Load ^1^	1.24 ± 0.36 (2.2–2.29)	2.95 ± 0.53 (3.84–4.07)	1.78 ± 0.46 (2.7–2.86)	3.19 ± 0.60 (4.07–4.31)	556.308	<0.001 ^ABCDEF^	0.760
Depression ^2^	1.11 ± 0.30 (1.07–1.15)	1.73 ± 0.53 (1.61–1.84)	2.06 ± 0.54 (1.96–2.15)	3.00 ± 0.66 (2.86–3.13)	379.352	<0.001 ^ABCDEF^	0.683
Anxiety ^3^	1.23 ± 0.35 (1.18–1.27)	1.63 ± 0.52 (1.52–1.75)	2.31 ± 0.60 (2.20–2.42)	3.23 ± 0.57 (3.12–3.34)	435.818	<0.001 ^ABCDEF^	0.713

* CI = confidence interval. # df = degrees of freedom. ^‡^ Tukey’s post hoc tests conducted for all the pairwise comparisons when the main effects were significant (i.e., *p* < 0.05). Letters following the main effect values indicate significant (*p* < 0.01) pairwise differences: ^A^ = Clusters 1 and 2 differed significantly; ^B^ = Clusters 1 and 3 differed significantly; ^C^ = Clusters 1 and 4 differed significantly; ^D^ = Clusters 2 and 3 differed significantly; ^E^ = Clusters 2 and 4 differed significantly; ^F^ = Clusters 3 and 4 differed significantly. ^1^ Cohen’s Perceived Stress Scale: the 2-item Stress Load subscale was used to assess maternal stress load. The 4-point-Likert-type scale: not at all, several days, more than half the days, nearly every day; scored from 1 to 4, respectively; higher scores indicate greater stress [15]. Cronbach’s alpha: 0.84. ^2^ The Patient’s Health Questionnaire-2 assessed maternal depression severity. The 4-point-Likert-type scale: not at all, several days, more than half the days, nearly every day; scored from 1 to 4, respectively; higher scores indicate greater depression [59,60,61]. Cronbach’s alpha: 0.81. ^3^ The Generalized Anxiety Disorder-2 assessed maternal anxiety. The 4-point-Likert-type scale: not at all, several days, more than half the days, nearly every day; scored from 1 to 4, respectively; higher scores indicate greater anxiety [62]. Cronbach’s alpha: 0.85.

**Table 2:** Sociodemographic Characteristics by Maternal Mental Health Status Cluster (N = 531).

Characteristic	Clusters				F Value df = 3, 527 #	ANOVA ^‡^ *p*	Partial Eta-Squared
	Cluster 1 Mean ± SD (CI *) n = 230	Cluster 2 Mean ± SD (CI) n = 83	Cluster 3 Mean ± SD (CI) n = 118	Cluster 4 Mean ± SD (CI) n = 100			
Maternal Age (years)	38.43 ± 5.82 (37.67, 39.18)	37.37 ± 5.66 (36.14, 38.61)	37.37 ± 5.66 (36.14, 38.61)	35.90 ± 6.00 (34.71, 37.09)	4.546	0.004 ^C^	0.025
Maternal Race/Ethnicity ^1^	0.50 ± 0.50 (0.44, 0.57)	0.60 ± 0.49 (0.49, 0.71)	0.42 ± 0.49 (0.33, 0.51)	0.60 ± 0.49 (0.50, 0.70)	3.448	0.017 ^DF^	0.019
Maternal Education Level ^2^	2.37 ± 0.75 (2.27, 2.47)	2.47 ± 0.74 (2.31, 2.63)	2.22 ± 0.73 (2.09, 2.35)	2.14 ± 0.74 (1.99, 2.29)	4.096	0.007 ^CE^	0.023
Parents in Household ^3^	0.83 ± 0.37 (0.79, 0.88)	0.80 ± 0.41 (0.71, 0.88)	0.74 ± 0.44 (0.66, 0.82)	0.73 ± 0.45 (0.64, 0.82)	0.382	0.077	0.012
Children ≤ 18 years in Household	2.29 ± 1.15 (2.14, 2.44)	2.14 ± 0.83 (1.96, 2.33)	2.10 ± 0.98 (1.92, 2.28)	2.13 ± 1.05 (1.92, 2.34)	1.086	0.355	0.006
Food Insecurity Risk ^4^	1.55 ± 0.75 (1.45, 1.65)	2.31 ± 0.87 (2.12, 2.50)	1.96 ± 0.88 (1.80, 2.12)	2.94 ± 0.86 (2.77, 3.11)	70.947	<0.001 ^ABCDEF^	0.288

* CI = confidence interval. # df = degrees of freedom. ^‡^ Tukey’s post hoc tests conducted for all the pairwise comparisons when the main effects were significant (i.e., *p* < 0.05). Letters following the main effect values indicate significant (*p* < 0.01) pairwise differences: ^A^ = Clusters 1 and 2 differed significantly; ^B^ = Clusters 1 and 3 differed significantly; ^C^ = Clusters 1 and 4 differed significantly; ^D^ = Clusters 2 and 3 differed significantly; ^E^ = Clusters 2 and 4 differed significantly; ^F^ = Clusters 3 and 4 differed significantly. ^1^ White = 0, non-White = 1. ^2^ On the 3-point scale: high school or less, some college, college graduate or higher; scored from 1 to 3, respectively; higher scores indicate greater educational attainment. ^3^ Single parent = 0, dual parent = 1. ^4^ On the 4-point Likert scale: definitely false, mostly false, mostly true, definitely true; scored from 1 to 4, respectively; higher scores indicate greater food insecurity [65].

**Table 3:** Weight-Related Parenting Cognitions by Maternal Mental Health Status Cluster (N = 531).

Parenting Cognitions ^1^	Clusters				F Value df = 3, 527 #	ANOVA ^‡^ *p*	Partial Eta-Squared
	Cluster 1 Mean ± SD (CI *) n = 230	Cluster 2 Mean ± SD (CI *) n = 83	Cluster 3 Mean ± SD (CI *) n = 118	Cluster 4 Mean ± SD (CI *) n = 100			
Self-Efficacy for Promoting Healthy Child Weight-Related Behaviors	4.28 ± 0.57 (4.21, 4.35)	4.04 ± 0.60 (3.91, 4.17)	4.04 ± 0.51 (3.95, 4.14)	3.92 ± 0.68 (3.78, 4.05)	10.950	<0.001 ^ABC^	0.059
Importance Placed on Modeling Healthy Weight-Related Behaviors for Children	4.16 ± 0.67 (4.07, 4.25)	3.94 ± 0.64 (3.80, 4.08)	3.89 ± 0.68 (3.77, 4.02)	3.83 ± 0.74 (3.68, 3.98)	7.471	<0.001 ^BC^	0.041
Importance Placed on Healthy Weight-Related Behaviors for Children	4.25 ± 0.54 (4.18, 4.32)	4.02 ± 0.50 (3.91, 4.13)	4.05 ± 0.50 (3.95, 4.14)	3.95 ± 0.54 (3.84, 4.06)	10.168	<0.001 ^ABC^	0.055
Encouragement and Facilitation of Children’s Healthy Weight-Related Behaviors	4.16 ± 0.58 (4.08, 4.23)	3.90 ± 0.64 (3.76, 4.04)	3.93 ± 0.49 (3.84, 4.02)	3.83 ± 0.59 (3.71, 3.95)	10.18	<0.001 ^ABC^	0.055
Perception that Engaging in Healthy Weight-Related Parenting Behavior is Worth the Effort	4.35 ± 0.69 (4.26, 4.44)	3.81 ± 0.99 (3.60, 4.03)	4.09 ± 0.71 (3.96, 4.22)	3.31 ± 1.16 (3.08, 3.54)	36.795	<0.001 ^ABCEF^	0.173
Family Support for Healthy Weight-Related Behaviors	4.00 ± 0.90 (3.88, 4.12)	3.32 ± 0.95 (3.11, 3.52)	3.73 ± 0.81 (3.58, 3.87)	2.83 ± 1.01 (2.63, 3.03)	42.359	<0.001 ^ABCDEF^	0.194

* CI = confidence interval. # df = degrees of freedom. ^‡^ Tukey’s post hoc tests conducted for all the pairwise comparisons when the main effects were significant (i.e., *p* < 0.05). Letters following the main effect values indicate significant (*p* < 0.01) pairwise differences: ^A^ = Clusters 1 and 2 differed significantly; ^B^ = Clusters 1 and 3 differed significantly; ^C^ = Clusters 1 and 4 differed significantly; ^D^ = Clusters 2 and 3 differed significantly; ^E^ = Clusters 2 and 4 differed significantly; ^F^ = Clusters 3 and 4 differed significantly. ^1^ For the 5-point agreement rating: strongly disagree, disagree, neutral, agree, strongly agree; scored from 1 to 5, respectively; all items in a scale are averaged to create the scale score; higher scores indicate a greater expression of the trait [52]. Cronbach’s alpha and the total number of items for the scales in the order displayed: 0.91 (13 items); 0.86 (7 items); 0.87 (13 items); 0.84 (12 items); 0.97 (12 items); and 0.94 (11 items).

**Table 4:** Home Food Environment by Maternal Mental Health Status Cluster (N = 531).

Characteristic	Clusters				F Value df = 3, 527 #	ANOVA ^‡^ *p*	Partial Eta-Squared
	Cluster 1 Mean ± SD (CI *) n = 230	Cluster 2 Mean ± SD (CI *) n = 83	Cluster 3 Mean ± SD (CI *) n = 118	Cluster 4 Mean ± SD (CI *) n = 100			
Child Feeding Practices ^1^ [52]							
Pressuring Children to Eat Nutrient-Dense Food	2.12 ± 1.01 (1.99, 2.25)	2.86 ± 1.23 (2.59, 3.12)	2.45 ± 1.06 (2.26, 2.64)	3.27 ± 1.14 (3.04, 3.49)	29.074	<0.001 ^ABCDF^	0.142
Restricting Children’s Access to Low-Nutrient-Dense Foods	2.61 ± 1.12 (2.47, 2.76)	3.29 ± 0.93 (3.09, 3.49)	3.18 ± 1.00 (3.00, 3.36)	3.72 ± 0.93 (3.54, 3.91)	29.869	<0.001 ^ABCEF^	0.145
Using Instrumental Feeding Strategies	2.19 ± 1.08 (2.04, 2.33)	2.82 ± 1.16 (2.57, 3.07)	2.45 ± 0.91 (2.28, 2.62)	3.08 ± 1.20 (2.84, 3.31)	18.338	<0.001 ^ACF^	0.095
Food Availability							
Home Availability of Fruits and Vegetables (servings/person/day) [68,69,70]	5.37 ± 2.11 (5.10, 5.65)	4.98 ± 1.97 (4.55, 5.41)	4.86 ± 1.88 (4.52, 5.21)	4.45 ± 2.13 (4.03, 4.88)	5.075	0.002 ^C^	0.028
Home Availability of Sugar-Sweetened Beverages (servings/person/day) [52]	0.46 ± 0.73 (0.36, 0.55)	0.49 ± 0.77 (0.32, 0.66)	0.67 ± 0.93 (0.50, 0.84)	0.77 ± 0.93 (0.58, 0.95)	4.113	0.007 ^C^	0.023
Home Availability of Foods for Breakfast ^1^ [52]	4.60 ± 0.73 (4.51, 4.70)	4.33 ± 0.80 (4.15, 4.50)	4.47 ± 0.72 (4.33, 4.60)	4.16 ± 0.94 (3.97, 4.35)	8.294	<0.001 ^ACF^	0.045
Family Mealtime (meals/week) [52]							
Family Mealtime Frequency	14.27 ± 5.69 (13.53, 15.00)	11.95 ± 5.67 (10.71, 13.19)	13.06 ± 5.86 (11.99, 14.13)	12.28 ± 5.96 (11.10, 13.46)	4.747	0.003 ^AC^	0.026
Family Meals in Unhealthy Settings	1.63 ± 2.71 (1.28, 1.98)	2.98 ± 3.58 (2.19, 3.76)	1.94 ± 2.35 (1.51, 2.37)	4.29 ± 4.27 (3.44, 5.14)	18.499	<0.001 ^ACEF^	0.095

* CI = confidence interval. # df = degrees of freedom. ^‡^ Tukey’s post hoc tests conducted for all the pairwise comparisons when the main effects were significant (i.e., *p* < 0.05). Letters following the main effect values indicate significant (*p* < 0.01) pairwise differences: ^A^ = Clusters 1 and 2 differed significantly; ^B^ = Clusters 1 and 3 differed significantly; ^C^ = Clusters 1 and 4 differed significantly; ^D^ = Clusters 2 and 3 differed significantly; ^E^ = Clusters 2 and 4 differed significantly; ^F^ = Clusters 3 and 4 differed significantly. ^1^ For the 5-point agreement rating: strongly disagree, disagree, neutral, agree, strongly agree; scored from 1 to 5, respectively; all items in a scale are averaged to create the scale score; higher scores indicate a greater expression of the trait. Cronbach’s alpha and the total number of items for the Child Feeding Practices scales in the order displayed: 0.83 (2 items); 0.85 (2 items); and 0.84 (2 items) [52].

**Table 5:** Child Health and Weight-Related Behaviors by Maternal Mental Health Status Cluster (N = 531).

Characteristic	Clusters				F Value df = 3, 527 #	ANOVA ^‡^ *p*	Partial Eta-Squared
	Cluster 1 Mean ± SD (CI *) n = 230	Cluster 2 Mean ± SD (CI *) n = 83	Cluster 3 Mean ± SD (CI *) n = 118	Cluster 4 Mean ± SD (CI *) n = 100			
General Health Rating ^1^ [71]	4.34 ± 0.75 (4.25, 4.44)	4.33 ± 0.78 (4.15, 4.50)	4.09 ± 0.82 (3.94, 4.24)	4.07 ± 0.98 (4.16, 4.30)	4.224	0.006 ^BC^	0.023
Physical Health Quality of Life (days/month) [71]	0.75 ± 3.22 (0.33, 1.17)	1.18 ± 3.26 (0.47, 1.89)	1.48 ± 4.25 (0.71, 2.26)	1.62 ± 2.64 (1.10, 2.14)	2.106	0.099	0.012
Mental Health Quality of Life (days/month) [71]	1.43 ± 2.19 (1.14, 1.71)	1.86 ± 1.75 (1.47, 2.24)	3.75 ± 6.35 (2.59, 4.90)	3.56 ± 4.55 (2.66, 4.46)	12.780	<0.001 ^BCDE^	0.068
BMI Percentile	54.17 ± 29.22 (46.55, 61.78)	45.05 ± 24.49 (33.59, 56.51)	55.63 ± 30.28 (44.71, 66.54)	60.32 ± 22.80 (49.33, 71.31)	1.041	0.377	0.024
Fruit and Vegetable Intake (servings/day) [68,72,73]	4.42 ± 1.64 (4.21, 4.63)	4.49 ± 1.39 (4.19, 4.79)	4.48 ± 1.49 (4.21, 4.75)	4.45 ± 1.88 (4.08, 4.82)	0.056	0.983	<0.001
Sugar-Sweetened Beverage Intake (servings/day) [52]	0.10 ± 0.14 (0.08, 0.12)	0.18 ± 0.30 (0.12, 0.25)	0.14 ± 0.15 (0.12, 0.17)	0.32 ± 0.37 (0.25, 0.39)	21.336	<0.001 ^ACEF^	0.108
Physical Activity Level ^2^ [74]	22.07 ± 12.96 (20.39, 23.76)	20.70 ± 11.91 (18.10, 23.30)	20.31 ± 11.67 (18.18, 22.43)	20.20 ± 11.56 (17.91, 22.49)	0.857	0.463	0.005
Sleep Duration (hours/day) [75,76]	9.01 ± 2.44 (8.70, 9.33)	8.51 ± 3.11 (7.83, 9.18)	8.41 ± 2.61 (7.93, 8.88)	7.45 ± 2.55 (6.94, 7.96)	8.391	<0.001 ^CEF^	0.046
Sleep Quality ^3^ [75,76]	4.55 ± 0.57 (4.47, 4.62)	4.34 ± 0.80 (4.16, 4.51)	4.34 ± 0.60 (4.23, 4.45)	4.28 ± 0.83 (4.12, 4.44)	5.250	0.001 ^BC^	0.029

* CI = confidence interval. # df = degrees of freedom. ^‡^ Tukey’s post hoc tests conducted for all the pairwise comparisons when the main effects were significant (i.e., *p* < 0.05). Letters following the main effect values indicate significant (*p* < 0.01) pairwise differences: ^A^ = Clusters 1 and 2 differed significantly; ^B^ = Clusters 1 and 3 differed significantly; ^C^ = Clusters 1 and 4 differed significantly; ^D^ = Clusters 2 and 3 differed significantly; ^E^ = Clusters 2 and 4 differed significantly; ^F^ = Clusters 3 and 4 differed significantly. ^1^ Centers for Disease Control Health-Related Quality of Life Scale 5-point excellence rating: poor, fair, good, very good, excellent; scored from 1 to 5, respectively; a higher score indicates better health [71]. ^2^ For the 7-point exercise days/week: 0, 1, 2, 3, 4, 5, 6, and 7; days/week are weighted by the exercise intensity, exercise level (weights of 1, 2, and 3 for walking, moderate, and vigorous activity, respectively) and summed to create a scale score; a higher scale score indicates a greater activity level; scores can range from 0 to 42 [74]. ^3^ Pittsburgh Sleep Quality Index 5-point rating scale: very bad, bad, okay, good, and very good; scored from 1 to 5, respectively, with higher scores indicating better sleep quality [75,76].

## Data Availability

Data sharing not applicable. No new data were created or analyzed in this study.
